# Supplementary material for: Comparison of analgesic activities of aconitine in different mice pain models
Source: PLoS One. 2021 Apr 1;16(4):e0249276. doi: 10.1371/journal.pone.0249276 (PMC8016268; doi:10.1371/journal.pone.0249276)
Supplement: Attachment — Submitted filename: Response to Reviewers.docx [file pone.0249276.s002.docx]

Mar 13th, 2021

John M. Streicher, Ph.D.
Academic Editor
PLOS ONE

Dear Dr. Streicher,

Thank you very much for your consideration of our manuscript entitled **“*Comparison of Analgesic Activities of Aconitine in Different Mice Pain Models*” (****ID: PONE-D-20-32048)** to be published in ***PLOS ONE***. Also, we gratefully appreciate for all of the suggestions and comments from reviewers. Those comments are all valuable and very helpful for revising and improving our paper. We have studied comments carefully and have made corrections which we hope meet with approval. We have modified the article in revisions mode. Our point-to-point responses to editor and the reviewers’ comments are shown below. We hope that the revised paper is sufficient for publication in ***PLOS ONE***.

Best wishes!

Regards,

Qian Feng, Ph.D.

International Institute for Translational Chinese Medicine,

Guangzhou University of Chinese Medicine,

Guangzhou, 510006, China

Tel: +86-020-39358071; Email: fengqian@gzucm.edu.cn

**Comments from the reviewers:**

-**Reviewer 1**

The manuscript has been improved in the revised version. However, several major issues remain to be improved.

Major Compulsory Revisions

(1) The authors only changed the format of the abstract but not really address the comments brought up in the primary evaluation.

**Response:** Thank you so much for your great efforts in improving the quality of our manuscript. The “Abstract” section has been re-written in our revised manuscript (Line 23-43).

(2) Introduction: This section is still not logically described and needed to be improved.

**Response:** Very appreciated for your important advice. The “Introduction” section has been reorganized in our revised manuscript (Line 45-99).

(3) Methods: The “model” group is the same treatment as the “control” group based on the information in Line 137-138 and Line 147-148.

**Response:** Thank you for your patience and carefulness on our manuscript. (1) In formalin induced nociception assay, mice were randomly divided into high dosage AC, low dosage AC, aspirin, model and control groups, with 6 mice in each group. The mice of high dosage AC, low dosage AC and aspirin groups were administered with 0.9 mg/kg AC, 0.3 mg/kg AC and 200 mg/kg aspirin respectively, while the mice of model and control groups were treated with 0.9% saline. One or two hours after administration, 20 μL of 0.92% formaldehyde solution was subcutaneously injected into the dorsal surface of the right hindpaw of the mice of high dosage AC, low dosage AC, aspirin and model groups, expect that the control group was treated with 0.9% saline.

(2) In CFA induced nociception assay, mice were randomly divided into high dosage AC, low dosage AC, aspirin, model and control groups, with 6 mice in each group. 20 μL of CFA was subcutaneously injected into the dorsal surface of the right hindpaw of the mice of high dosage AC, low dosage AC, aspirin and model groups, while the control group was treated with 0.9% saline instead. After 48 hours, the mice of high dosage AC, low dosage AC and aspirin groups were administered with 0.9 mg/kg AC, 0.3 mg/kg AC and 200 mg/kg aspirin respectively，and the mice of model and control groups were treated with 0.9% saline.

(4) Results: The low dose of AC showed the most significant improvement based on the images of the hind paw in Figure 2. However, the bar graph doesn’t indicate that

**Response:** Thank you very much for your constructive question. The swelling improvement effect of the 0.3mg/kg AC group was similar to that of the aspirin group, but better than the 0.9mg/kg AC group, which may be due to individual differences. At present, we do not know why low dosage AC was better than high dosage AC after long time treatment. We will further study the analgesic and anti-swelling effects of AC.

(5) The English language issue still needs to be improved throughout the whole manuscript.

**Response:** Many thanks for your great efforts in improving the quality of our manuscript. The manuscript has been further edited by highly qualified native English speakers.

**-Reviewer 2**

The authors have appropriately addressed my original comments but the revised version has a few things that need to be addressed.

(1) In the abstract (line 27) the authors state they are looking at the analgesic activities of AC on neuropathic pain. However, they do not use a neuropathic pain model. This should be revised to say they are looking at the analgesic effects of AC on thermal sensitivity, or something similar.

**Response:** Thank you so much for your constructive suggestion. The description about “neuropathic pain” has been revised to “acute thermal stimulus pain” (Line 34).

2. Similarly, in the abstract (line 38) they state AC had analgesic effect on neuropathic pain but they only use the hot plate to measure thermal sensitivity in naive mice, not a neuropathic pain model. Please revise this sentence as well.

**Response:** Thank you so much for your carefulness on our manuscript. This sentence has been revised in Line 34.

3. The use of the word 'videlicet' in line 169 does not seem appropriate

**Response:** Very appreciated for your great efforts in improving the quality of our manuscript. We have rewritten the sentence in Lin 176-178.

4. The sentence ending on Line 236 would make more sense if it read '48 hours after CFA injection' instead of 'after CFA injection 48 hours'

**Response:** Many thanks for your helpful suggestion. We have modified the expression in Line 239.

5. The conclusion drawn in lines 236-238 is not accurate unless the different time points following CFA injection were compared statistically. From figure 2, it does not appear that the different time points were compared, only the different groups.

**Response:** Thank you very much for your important question. Indeed, we didn’t compare the different time points in Figure 2. The conclusion in Line 236-238 has been revised.

6. The wording of the conclusion on line 267 that AC was able to relieve neuropathic pain is inappropriate (similar to comments 1 and 2). This should say thermal sensitivity or something similar.

**Response:** Thanks very much for improving the quality of our manuscript. The description has been revised to “acute thermal stimulus pain” (Line 269).

7. Lines 282-283 also mention AC having an effect on neuropathic pain

**Response:** Thank you again for your important suggestion. The description has been revised in Line 284-286.

8. In Table 3 for the CFA induced nociception assay, what does the 7th compare with 1st value mean?

**Response:** Thank you for your constructive question. In CFA induced nociception assay, drug-treated groups were orally given AC or aspirin respectively once a day for seven consecutive days. Compared with day 1, the improvement rate of pain threshold on day 7 reflected the analgesic effect of aspirin and AC after the whole treatment cycle. Importantly, the improvement of pain threshold in low dosage AC group was similar to that of aspirin group, which indicating that AC treatment showed powerful inhibitory effect on inflammatory pain.
